# Cost-Effectiveness of a Short Message Service Intervention to Prevent Type 2 Diabetes from Impaired Glucose Tolerance

**DOI:** 10.1155/2016/1219581

**Published:** 2015-12-21

**Authors:** Carlos K. H. Wong, Fang-Fang Jiao, Shing-Chung Siu, Colman S. C. Fung, Daniel Y. T. Fong, Ka-Wai Wong, Esther Y. T. Yu, Yvonne Y. C. Lo, Cindy L. K. Lam

**Affiliations:** ^1^Department of Family Medicine and Primary Care, The University of Hong Kong, Ap Lei Chau, Hong Kong; ^2^Department of Medicine and Rehabilitation, Tung Wah Eastern Hospital, Causeway Bay, Hong Kong; ^3^School of Nursing, The University of Hong Kong, Pokfulam, Hong Kong

## Abstract

*Aims*. To investigate the costs and cost-effectiveness of a short message service (SMS) intervention to prevent the onset of type 2 diabetes mellitus (T2DM) in subjects with impaired glucose tolerance (IGT). *Methods*. A Markov model was developed to simulate the cost and effectiveness outcomes of the SMS intervention and usual clinical practice from the health provider's perspective. The direct programme costs and the two-year SMS intervention costs were evaluated in subjects with IGT. All costs were expressed in 2011 US dollars. The incremental cost-effectiveness ratio was calculated as cost per T2DM onset prevented, cost per life year gained, and cost per quality adjusted life year (QALY) gained. *Results*. Within the two-year trial period, the net intervention cost of the SMS group was $42.03 per subject. The SMS intervention managed to reduce 5.05% onset of diabetes, resulting in saving $118.39 per subject over two years. In the lifetime model, the SMS intervention dominated the control by gaining an additional 0.071 QALY and saving $1020.35 per person. The SMS intervention remained dominant in all sensitivity analyses. *Conclusions*. The SMS intervention for IGT subjects had the superiority of lower monetary cost and a considerable improvement in preventing or delaying the T2DM onset. This trial is registered with ClinicalTrials.gov NCT01556880.

## 1. Introduction

Prevention of diabetes mellitus (DM) is becoming an increasing urgent public health concern all over the world. It is estimated that there are 382 million diabetic subjects by 2013 in the world, and the number will increase to 592 million by 2035 [1]. The rising prevalence of DM and its devastating complications [2] poses a huge threat to human health and places enormous economic burden to the society [3, 4]. Studies indicated that prediabetes is a major factor leading to diabetes [5], and people with prediabetes have increased risk of cardiovascular disease (CVD) and mortality [6–8].

Given the potentially immerse disease burden caused by prediabetes, it is impending to implement cost-effective interventions to delay diabetes among subjects with prediabetes and even reverse the prediabetes status. Delivery of short message service (SMS) is part of mobile-based interventions that has bridged an effective communication channel to endeavor behavioral change, enhance disease-specific knowledge, and subsequently improve health outcomes in the field of preventive medicine and chronic disease self-management. Over the past decade, self-management education or support by mobile-based applications has been launched to target the blood glucose and HbA1C levels control in patients with type 2 diabetes mellitus (T2DM) [9]. Mobile-based applications are evident to modify the individual behavior to quit smoking [10], but they are rarely applied in diabetes prevention. A randomized controlled trial was conducted recently to evaluate the efficacy of the SMS intervention and it showed encouraging results in reducing T2DM onset among patients with impaired glucose tolerance (IGT) [11].

Regarding the cost-effectiveness analyses for add-on interventions conventionally applied to prediabetes, the prediabetic screening [12] and lifestyle modification [13–16] have been demonstrated to be highly cost-effective strategies in comparison with usual clinical practice. To determine whether a lifestyle modification is cost-effective, it depends on how preventive the specific strategy is and how much it costs. Although previous cost-effective strategies of lifestyle intervention have different combinations of interventions, they achieve cost-effectiveness outcomes by decreasing the incidence of T2DM and its complications through slowing the progression to T2DM and early detection of undiagnosed abnormal glucose tolerance [16–19].

In view of the significant effectiveness of the SMS intervention in preventing T2DM among prediabetes subjects and its relative low cost, it is tempting to assume that the SMS intervention is cost-effective. However, it is unethical and infeasible to wait until perfect lifetime data are available to validate the cost-effectiveness of any intervention. Decision modelling is proved to be an effective method to conduct economic evaluation of clinical interventions over lifetime. This technique compares the intervention of interest with the alternative options by incorporating all appropriate evidence of costs and effectiveness, simulating disease paths over longer time span, and reflecting uncertainty in evidence [20].

Using data from relevant epidemiological studies and clinical trials, we constructed a Markov model to compare the strategy of delivering the SMS intervention programme as an additional support to usual clinical practice and usual clinical practice alone in managing subjects with IGT. Depending upon the strategy applied, subjects in SMS intervention group had lower T2DM onset rate compared to the usual care, resulting in lower cost of managing T2DM and longer life years, which might compensate the additional cost of delivering the SMS intervention.

## 2. Method

### 2.1. Markov Model

**Figure 1:**
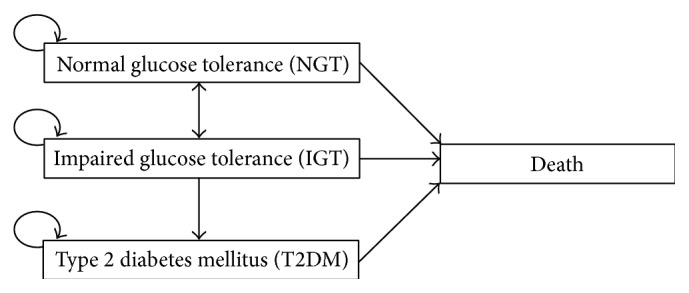
Annual transition diagram of Markov model.

A decision analytic model with a state-transition Markov process [21] was developed to simulate long-term effects of cost and clinical effectiveness of interventions in a cohort of prediabetes under two main strategies, which were (1) SMS intervention in addition to usual clinical practice and (2) usual clinical practice. The TreeAge Pro 2013 software (TreeAge Software, Inc., Williamstown, MA, USA) was used for the modelling. Long-term modelling referred to time horizon over a 50-year period beyond the two-year intervention. The natural history referred to previous economic evaluation of diabetes prevention [15, 22–24]. The one-year transition cycle moved from one health state to another amongst four Markov states: normal glucose tolerance (NGT), IGT, T2DM, and death (Figure 1). An individual had the likelihood to transit from current health state to a different health state or remain in their current health state at the end of one-year cycle in this Markov process [21]. Compared to the usual practice group, the SMS intervention led to different transition probabilities among these four disease states, resulting in extra cost in addition to usual care. For patients who transit to diabetes were assumed to stop receiving SMS intervention, therefore, the management of T2DM in these two groups was assumed to follow the same routine clinical practice in primary care setting. In other words, the costs and health effects of T2DM were the same for all subjects, regardless of which group they belonged to. An annual discount rate of 3% was undertaken in both the cost and health outcomes as per the Panel on Cost-Effectiveness in Health and Medicine recommended [25].

### 2.2. Transition Probability

As shown in Table 2, the annual transition probabilities between health states were taken from several data sources, including epidemiological studies and estimation from cost-effectiveness models. The annual transition probability from IGT to T2DM in the usual practice group in the first three years was adopted from the results of the placebo arm in Diabetes Prevention Program (DPP) [27], whereas the Diabetes Prevention Program Outcome Study (DPPOS) [28] provided this transition probability for the fourth year onwards. Subjects with IGT had about double risk for T2DM during the first three years compared to that in the fourth year onwards [27, 28]. The effect of SMS on the transition from IGT to T2DM was reflected in the relative risk of T2DM onset for SMS intervention against control groups, which was reported by a randomized control trial (Clinical Trials Registry Number: NCT01556880) among a sample of Chinese professional drivers with IGT [11, 29–31]. We adopted the relative risks for complete case analysis reported. This trial also reveals the drop-out rates of SMS intervention during the first year and second year, which were 38.9% and 30.3%, respectively. The proportion of subjects who regressed to normal glucose regulation was taken from the Caro et al. study [22] which was used to derive the annual transition probabilities from DPP. Subjects with diagnosed T2DM would either stay in that state or be absorbed into the death state in the next year. All-cause mortality rates for NGT were adopted from the Hong Kong Life Table 2011 [32]. The relative risks of mortality in IGT and T2DM were 1.50 (95% CI 1.10–2.00) and 2.30 (95% CI 1.60–3.20), respectively, which were used to adjust the age-specific death rate for subjects with IGT or T2DM [23]. The incidence and mortality rates reported in literature were converted to annual transition probabilities using mathematical formula [33]: *p* = 1 − exp⁡(−*rt*), where *p* is the transition probability, *r* is the rate, and *t* is the unit of time. Although subjects with diabetic complication had a higher risk of mortality, our model did not differentiate different complications related to diabetes and therefore applied the average mortality of DM for all diabetic subjects.

### 2.3. Costs

Costs were estimated from the perspective of health service provider. No clinical health service was deployed to routinely screen or treat patients with prediabetes in Hong Kong, and thus there were no costs assigned to patients with NGT or prediabetes in the usual practice group. The cost of a SMS intervention was the sum of two components: the delivery cost of a total of 66 text messages package via online programme platform and the staff cost of sending those text messages to each subject. The delivery cost of text messages per subject was $4.15 in first year and $0.92 in second year, in total of $5.08, while the staff cost of sending text messages was estimated as $36.95 based on an estimate of 5 minutes for delivering one SMS online and a median hourly wage of $6.72 in 2011 [26]. The total intervention cost per subject was $42.03. All costs were calculated in 2011 Hong Kong dollars and converted to US dollars at a pegged rate of US$1 = HKD$7.8. The annual total medical costs attributed to each patient with type 2 diabetes were $1492.05 in 2004 year price [3], and those costs incurred in the usual clinical practice were inflated to $1,729.90 in 2011 using the medical services price index taken from Hong Kong Census and Statistics Department [26]. The medical costs associated with diabetes management were assumed to be the same for all the years in this state. All costs data are summarized in Table 1.

### 2.4. Utilities

To calculate the quality adjusted life years (QALYs) accumulated in the lifetime model, we adopted the utility scores for each health state from literature. We applied the same utility scores for subjects with NGT and IGT. The utility scores for NGT/IGT and T2DM were 0.76 and 0.72, respectively [23].

### 2.5. Cost-Effectiveness Outcomes

The main outcomes of the cost-effectiveness analysis in this study were the incremental cost-effectiveness ratios (ICER), in terms of cost per event (T2DM onset) prevented, cost per life year gained, and cost per QALY gained. The SMS intervention dominated control group if the control group was more expensive and less effective.

### 2.6. Sensitivity Analysis

Sensitivity analysis was performed to explore the uncertainty on the clinical and interventional parameters of model. Sensitivity analysis for the ICER of the SMS intervention compared with usual clinical practice was conducted on the transition probabilities with ranges suggested by previous literature and experts in family medicine and endocrine (Table 2). In addition, annual discount rate with limits bounded by 0% (undiscounted) and 5% was included in the analysis. Threshold analysis was undertaken to capture the threshold values of model interventional parameters at which the 50-year cumulative costs of SMS group were equivalent to those of control group. For instance, threshold values of intervention costs guided decision making on the level of intervention subsidy from the health service provider.

## 3. Results

### 3.1. Base-Case Scenario

The base-case scenario was based on the SMS intervention and T2DM costs from Table 1 and previously reported clinical parameters values from Table 2. Results of base-case scenario are shown in Table 3. During the two-year SMS intervention, compared to the usual practice group, each subject in SMS group costs $118.39 less and the SMS group reduced 5.05% of T2DM onset. Over the 50-year period of time horizon, the cumulative costs of the SMS intervention group per subject were substantially lower than the control group. Given the 0.063 life years and 0.071 QALYs gained in SMS group, the SMS intervention was a less costly but more effective strategy than control group. As a result, the SMS intervention was beneficial for effectiveness and cost-saving compared with the usual clinical practice in both short and long time horizons.

### 3.2. Sensitivity Analysis

Sensitivity analysis was performed by varying clinical and interventional parameters to test the robustness of model conclusion.  Table 4 presents the results of sensitivity analysis which assessed the model robustness of base-case scenario. The determinant status of SMS group stayed unchanged when we varied various key parameters including SMS drop-out rates, annual transition probabilities between health states, and discount rate. Increased drop-out rate at first year from base-case value to 100% led to the zero increment cost, remaining dominant. Despite variation in drop-out rates, the SMS intervention dominated control group. When the annual transition probability from IGT to T2DM at the first year of SMS intervention was increased from 3.53% to 11.57%, which is equal to that in the control group, the SMS group still cost $76.21 less, remaining dominant. The threshold analysis, as shown in Table 5, indicated that SMS group reached the equivalent amount of total costs of control groups when the first year intervention cost was increased to $1,704.04 per person from original cost of $34.38. When the first year intervention cost was fixed at $34.38, to reach the same total cost as control group, the SMS intervention cost in the second year should have increased from $7.64 to $3,093.78. The annual transition probability from IGT to T2DM in first year in SMS group had to rise from 3.53% to 12.22% before the SMS group became no cheaper than control group. Similarly, only if the annual probability from IGT to T2DM in second year enrolled in SMS increased to 22.60% or higher, three times as base-case scenario, would their total medical cost be higher than the subjects in control group.

## 4. Discussion

This study found that, compared to usual clinical practice only, it was cost-saving to add the nonpharmacological SMS intervention in both the short and long time horizon. Given the high drop-out rate in the SMS group from the previous study, we assumed that the SMS intervention was provided only in the first two years. Within the two-year span, the SMS intervention led to a reduction of 5.05% of diabetes onset in comparison with the usual practice. Although it costs a total of $42.03 per subject to conduct the SMS intervention for two years, the money saved by treating less diabetic cases overcompensated the cost of SMS intervention. As a result, the SMS group cost $118.39 less per subject and prevented 5.05% of diabetes onset. When projecting the effects of the discrepancy in DM onset between the two groups to the lifetime span, we found that the SMS intervention accrued more life years gained (incremental effectiveness: 0.071 QALY) and less cost (incremental cost: −$1,020.35) than the control group. The robustness of the lifetime model was verified by sensitivity analysis through varying the transition probabilities and drop-out within wide ranges. The threshold analysis showed that only when the cost of SMS intervention climbed to about $2000 or more to reach the break-even point with the control group.

While the SMS intervention has been shown to be cost-saving in prediabetic population, the ICER values were not available as the incremental cost is negative. Using the empirical DPP/DPPOS data [16], the 10-year cost-effectiveness analysis estimated that metformin overwhelmed the control group with direct medical cost of care, and the ICER for lifestyle intervention compared to control group was $10,037 per QALYs gained. Complementary results were reported in Australian study modelling from a third-party payer perspective [23], indicating that the intensive lifestyle modification dominated the control group but the ICER for the metformin versus control group was $10,142 in Australian dollars per QALYs gained. Lifestyle interventions in IGT were found cost-effective in other contexts as well, while most of these studies also adopted the effectiveness data from the DPP/DPPOS cohort [22, 34, 35]. Some studies on the CEA of interventions in prediabetes have established more sophisticated models, which involve development of complications after subjects developed diabetes. These studies adopted the Framingham or UKPDS risk functions to differentiate the risks of diabetic complications in long term based on the changes of clinical parameters (i.e., HbA1c, lipid profile, systolic blood pressure, etc.) observed in the trials. The incremental effectiveness in these studies is more prominent than the finding in our study that 0.14 to 0.50 life years were gained by lifestyle intervention compared to no intervention over the lifetime [14, 18]. Although the incremental life years and QALYs found in our model were smaller, it is noteworthy that we made a rather conservative estimation, as we assumed the two intervention groups have the same utilities after they developed diabetes. Moreover, we found that the SMS intervention is cost-saving over both short term and long term, which is favouring results for policy makers.

The main strength of this cost-effectiveness analysis model was that the sources of interventional parameter were the valid estimate reported in a randomized controlled trial. Additionally, our model estimated the short-term as well as long-term outcomes of cost-effectiveness. This is important as the initial setup cost invested to SMS group was shortly balanced out within two years, and the incremental cost was proportionally magnified between the two groups over a 50-year period. As the threshold analysis supported the increased costs of SMS in first and second year, there is potential area to set the patients' out-of-pocket charges of SMS intervention implemented in health service. Subsidy given by health service provider or government may be an alternative approach to deal with financing of the SMS implementation. Threshold analysis further offered cautionary advice on the termination of SMS intervention when the annual transition probabilities from IGT to T2DM in first and second year of SMS group exceeded 12.22% and 22.60%, respectively. The scenarios implied that the SMS intervention was no longer supported by the nature of cost-saving. The feasibility and sustainability of SMS intervention are another concern due to about one-third of drop-out rates in first and second year. Owing to the high levels of drop-out, the effects of SMS intervention on ICER were considerably diluted. In a scenario of 100% drop-out in second year participation as illustrated in sensitivity analysis, the incremental cost ($765.21) of one-year intervention was smaller than that in base-case scenario.

Several drawbacks were noted in this study. Firstly, the effectiveness of SMS intervention was based on a RCT in Hong Kong Chinese population, but some clinical and epidemiological data were adopted from DPP and DPPOS, which are the US population-based studies. Due to lack of Hong Kong local data, we assumed that data from other sources were applicable in our model. It is likely that there may be substantial differences in the clinical and epidemiological data between the Hong Kong and the US populations; however, the values of these parameters were applied the same in both SMS group and control group, which will not affect the relative effectiveness in the model. Secondly, the interventional parameters were derived from randomized controlled trial on the pilot basis with 104 IGT subjects participating. Although the intervention has been shown to be effective in pilot data, the effect of SMS on the reduction of T2DM onset over time may be diluted in population-based setting. This study reflects the reasonable need for undertaking the SMS intervention on mass population with prediabetes. Thirdly, the model was built based on some simplified assumptions. For example, annual cost of T2DM was uniform regardless of gender, complication experienced, and insulin treated. Given the more diabetic complications experienced, direct medical costs associated with a T2DM subject increased sharply [36]. Furthermore, the model did not account for the health states representing the presence of diabetic complications as no evidence available shows that the SMS intervention in prediabetes has impacts on the incidence of diabetic complications.

## 5. Conclusions

This cost-effectiveness analysis reveals that the SMS intervention for subjects with prediabetes had the superiority of lower cost and a considerable improvement in preventing or delaying the T2DM onset. Encouraging efforts of clinical and cost-effectiveness outcomes were diluted due to the loss of participation over the 2-year intervention. This study indicated that it was cost-saving to prevent T2DM through implementing nonpharmacological SMS intervention among prediabetics.

## The Significant Findings of the Study

The SMS intervention was a low-cost and effective programme for type 2 diabetes mellitus prevention in subjects with impaired glucose tolerance, resulting in cost-saving to health service provider regardless of 2-year trial and 50-year lifetime periods.

## What This Study Adds

This trial-based cost-effectiveness analysis showed that the SMS may be an add-on intervention applied to impaired glucose tolerance management in routine clinical practice in primary care setting.

## Figures and Tables

**Table 1:** Unit costs (US$, year 2011 values) for the SMS intervention.

Resource component	Unit cost	Reference
SMS intervention		
First year		
Delivery charges of SMS via online platform^*∗*^	$4.15	[11]
Staff wage for SMS delivery^†^	$30.23	[26]
Second year		
Delivery charges of SMS via online platform^*∗*^	$0.92	[11]
Staff wage for SMS delivery^†^	$6.72	[26]
Annual cost of T2DM^‡^	$1,727.90	[3]

*Note*. ^*∗*^A total of 66 short text messages' package is sent to each subject. ^†^An estimate of 5 minutes was spent for each SMS delivery and in total 330 minutes was used for each subject, assuming the median hourly wage of $6.72. ^‡^Annual cost in 2004 year price was inflated to 2011 year price using the medical services price index taken from Hong Kong Census Department.

**Table 2:** Clinical parameter value in base-case scenario and range used in sensitivity analysis.

Parameter	Base-case	Sensitivity analysis		Reference
		Minimum	Maximum	
SMS intervention				
Drop-out rate at year 1	38.89%	0%	100%	[11]
Drop-out rate at year 2	30.30%	0%	100%	[11]
Relative risk of T2DM at year 1 from IGT	0.34	0.10	1.00	[11]
Relative risk of T2DM at year 2 from IGT	0.60	0.10	1.00	[11]
Annual transition probability from IGT to NGT				
At all years	16.20%	5%	25%	[22]
Annual transition probability from NGT to IGT				
At all years	16.30%	5%	25%	[22]
Incidence rate (cases per 100 person-years) of T2DM from IGT				
Control at years 1–3	11.0	9.8	12.3	[27]
Control at year 4+	5.6	4.8	6.5	[28]
Relative risk of mortality				
IGT	1.5	1.1	2	[23]
T2DM	2.3	1.6	3.2	[23]
Utility				
NGT	0.76	NA		[23]
IGT	0.76	NA		[23]
T2DM	0.72	NA		[23]
Discount rate	3%	0%	5%	[25]

Note: NGT = normal glucose tolerance; IGT = impaired glucose tolerance; T2DM = type 2 diabetes mellitus; NA = not applicable.

**Table 3:** Results of base-case scenario.

Base-case scenario	SMS	Control	Incremental
2-year period			
Mean cost (in USD) accrued per patient	342.94	461.33	–118.39
T2DM onset	12.55%	17.60%	–5.05%
Cost per T2DM onset prevented			Dominance
50-year period			
Mean cost (in USD) accrued per patient	12107.40	12958.17	–850.77
LYs per patient	19.24	19.08	0.063
QALYs per patient	14.248	14.177	0.071
Cost per LY gained			Dominance
Cost per QALYs gained			Dominance

Note: T2DM = type 2 diabetes; LYs = life years; QALY = quality adjusted life year.

**Table 4:** Results of sensitivity analyses.

Parameters	Base-case	Range for sensitivity analysis	Range for incremental cost (USD)	Range for cost per LYs gained
SMS drop-out rate at year 1	38.89%	0.00%–100.00%	−1669.66 to 0.00	Dominance
SMS drop-out rate at year 2	30.30%	0.00%–100.00%	−1131.28 to −765.21	Dominance
Annual transition probability				
From IGT to T2DM, control at year 1–3	10.42%	9.34%–11.57%	−950.73 to −761.82	Dominance
From IGT to T2DM, SMS at year 1	3.53%	0.93%–11.57%	−1324.36 to −76.21	Dominance
From IGT to T2DM, SMS at year 2	6.25%	0.93%–11.57%	−1352.05 to −688.20	Dominance
From IGT to NGT	16.20%	5.00%–25.00%	− 922.28 to −1063.06	Dominance
From NGT to IGT	16.30%	5.00%–25.00%	−1117.45 to −979.19	Dominance
RR of mortality in IGT	1.5	1.1–2.0	−1015.49 to −1026.04	Dominance
RR of mortality in T2DM	2.3	1.6–3.2	−1087.30 to −953.95	Dominance
Discount rate	3.00%	0.00%–5.00%	−1450.41 to −836.95	Dominance

Note: NGT = normal glucose tolerance; IGT = impaired glucose tolerance; T2DM = type 2 diabetes mellitus; RR = relative risk.

**Table 5:** Threshold analysis of parameters at which the costs of SMS intervention and control became equivalent over a 50-year period.

Parameters	Base-case	Threshold
SMS intervention cost at year 1	$34.38	$1,704.04
SMS intervention cost at year 2	$7.64	$3,093.78
Annual transition probability		
From IGT to T2DM, SMS at year 1	3.53%	12.22%
From IGT to T2DM, SMS at year 2	6.25%	22.60%

Note: IGT = impaired glucose tolerance; T2DM = type 2 diabetes mellitus.

## Abbreviations

SMS: Short message service
T2DM: Type 2 diabetes mellitus
IGT: Impaired glucose tolerance
QALY: Quality adjusted life year
DPP: Diabetes Prevention Program
DPPOS: Diabetes Prevention Program Outcome Study
ICER: Incremental cost-effectiveness ratios.
